# Cytoadherence and virulence - the case of *Plasmodium knowlesi *malaria

**DOI:** 10.1186/1475-2875-11-33

**Published:** 2012-02-03

**Authors:** Farrah A Fatih, Angela Siner, Atique Ahmed, Lu Chan Woon, Alister G Craig, Balbir Singh, Sanjeev Krishna, Janet Cox-Singh

**Affiliations:** 1 Centre for Infection and Immunity, St George's University of London, London SW17 0RE, UK; 2 Malaria Research Centre, University Malaysia Sarawak, Kuching 93150, Malaysia; 3 Pathology Laboratory, Hospital Sarikei, Sarikei 96100, Malaysia; 4 Molecular and Biochemical Parasitology, Liverpool School of Tropical Medicine, Liverpool L3 5QA, UK

**Keywords:** *P. knowlesi*, Cytoadherence, SICAvar, ICAM-1, VCAM, CD36, Malaria, Coma

## Abstract

**Background:**

Cytoadherence of infected red blood cells to brain endothelium is causally implicated in malarial coma, one of the severe manifestations of falciparum malaria. Cytoadherence is mediated by specific binding of variant parasite antigens, expressed on the surface of infected erythrocytes, to endothelial receptors including, ICAM-1, VCAM and CD36. In fatal cases of severe falciparum malaria with coma, blood vessels in the brain are characteristically congested with infected erythrocytes. Brain sections from a fatal case of knowlesi malaria, but without coma, were similarly congested with infected erythrocytes. The objective of this study was to determine the binding phenotype of *Plasmodium knowlesi *infected human erythrocytes to recombinant human ICAM-1, VCAM and CD36.

**Methods:**

Five patients with PCR-confirmed *P. knowlesi *malaria were recruited into the study with consent between April and August 2010. Pre-treatment venous blood was washed and cultured *ex vivo *to increase the proportion of schizont-infected erythrocytes. Cultured blood was seeded into Petri dishes with triplicate areas coated with ICAM-1, VCAM and CD36. Following incubation at 37°C for one hour the dishes were washed and the number of infected erythrocytes bound/mm^2 ^to PBS control areas and to recombinant human ICAM-1 VCAM and CD36 coated areas were recorded. Each assay was performed in duplicate. Assay performance was monitored with the *Plasmodium falciparum *clone HB3.

**Results:**

Blood samples were cultured *ex vivo *for up to 14.5 h (mean 11.3 ± 1.9 h) to increase the relative proportion of mature trophozoite and schizont-infected red blood cells to at least 50% (mean 65.8 ± 17.51%). Three (60%) isolates bound significantly to ICAM-1 and VCAM, one (20%) isolate bound to VCAM and none of the five bound significantly to CD36.

**Conclusions:**

*Plasmodium knowlesi *infected erythrocytes from human subjects bind in a specific but variable manner to the inducible endothelial receptors ICAM-1 and VCAM. Binding to the constitutively-expressed endothelial receptor CD36 was not detected. Further work will be required to define the pathological consequences of these interactions.

## Background

Coma is one of the manifestations of *Plasmodium falciparum *malaria in children and adults [1,2] and it carries a poor prognosis. The accumulation of cytoadherent parasitized erythrocytes in post-capillary venules of the brain is strongly causally implicated in precipitating malarial coma [3-5]. Adherence to brain and other endothelial surfaces is mediated by the expression of variant parasite-derived proteins (Pf EMP1 *var *family) on the *P. falciparum *infected erythrocyte surface [6]. PfEMP1 proteins predominantly bind to CD36, but also to inducible Intercellular Adhesion Molecule 1 (ICAM-1) [7-10]. Binding to up-regulated ICAM-1 is particularly important in cytoadherence to brain endothelium because CD36 is not expressed in this endothelial compartment [8,9].

Malarial coma is rare in other infections by the human host-adapted *Plasmodium *species and coma has not been a feature of severe and fatal zoonotic *Plasmodium knowlesi *malaria [11-13]. However, *post mortem *examination of a fatal case of severe knowlesi malaria without coma showed brain capillaries and venules congested with infected erythrocytes [14]. Expressed parasite variant surface antigens had been described in experimental *P. knowlesi *infections of rhesus monkeys before PfEMP1 was identified in *P. falciparum *[15]. *Plasmodium knowlesi *surface proteins were named Schizont-Infected Cell Agglutination Antigens (SICA) and are encoded by the SICA*var *gene family [16]. Although distantly related, SICAvar proteins share binding signature motifs with PfEMP1 proteins [17]. The remarkable histological similarity between brain sections from fatal *P. knowlesi *malaria and fatal cases of severe falciparum malaria with coma [14,18], particularly the accumulation of infected erythrocytes in brain microvasculature, led to the design of this study to test the binding characteristics of *P. knowlesi *isolates from patients [9].

## Methods

### Patient recruitment

Patients with malaria admitted to Hospital Sarikei in Sarawak, Malaysian Borneo were recruited, with informed consent, into this study between April and August 2010. The study was approved by the Malaysian Ministry of Health Medical Research and Ethics Committee. Infecting species was confirmed by *Plasmodium *species-specific nested-PCR assays [19] and only patients with single species infections were retained in the study.

### Blood collection and ex vivo parasite development

**Figure 1:**
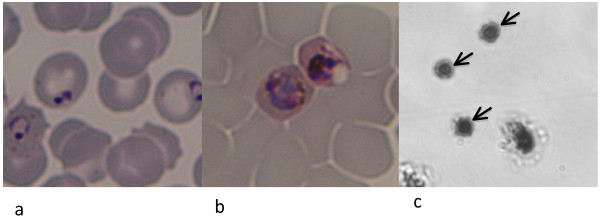
***Plasmodium knowlesi *static binding assay**. On admission to the study this patient had predominantly ring (immature trophozoite) stage parasites (**a**). Following a period of *in vitro *culture the parasites matured, in this case to late trophozoite stages, as required for the static binding assay (**b**). Infected erythrocytes bound to an ICAM-1 coated area of the assay dish are marked with arrows (**c**).

Approximately 2.5 mL of pre-treatment venous blood from each patient was collected into EDTA. RPMI 1640 medium supplemented with 20 mM D-glucose, 25 mM HEPES, 25 mg/ml gentamicin sulphate, 15% human AB plasma with 0.2 mM hypoxanthine was used for parasite culture. Gently washed loosely packed cells from each patient were re-suspended in culture medium to approximately 5% haematocrit and cultured at 37°C under 5% O_2_, 5% CO_2 _and 90% N_2_. Thin blood film microscopy was used to follow parasite development until at least half the parasites had developed into late trophozoites (parasites with dense cytoplasm and undivided nuclear chromatin mass) and schizonts (at least three divided nuclear chromatin masses with pigment granules). See Figure 1a and 1b.

### Static protein binding assays

A method adapted from McCormick *et al. *[9] to test the ability of infected erythrocytes to bind to purified recombinant human Fc chimera ICAM-1, VCAM, and CD36 (R&D Systems, Minneapolis, USA) was used. Three identical areas of each Petri dish (60 mm diameter, product code 351007, Becton, Dickenson and Company, NJ, USA) were treated with 2 μl aliquots of purified ICAM-1, VCAM, CD36, each at 100 μg/mL. Control areas were treated with phosphate buffered saline (PBS) and three marked areas were left untreated. The dishes were incubated in a humid chamber at 37°C for two hours before aspirating off excess protein and blocking all areas with 1% w/v bovine serum albumin in PBS for 2 h at 37°C. The blocking solution was removed by gentle pipetting. *Ex vivo *matured infected erythrocyte cultures were added to 3 ml warmed binding buffer (RPMI 1640 media supplemented with D-glucose) to a final haematocrit of 3%. Each protein and the PBS control were represented in triplicate per dish and duplicate dishes were seeded per patient isolate. Therefore there were six replicate areas per protein or PBS control for each patient sample assayed. Dishes were seeded with 1.5 ml of the prepared *ex vivo *cultured cell suspension per patient isolate and a third assay dish with *P. falciparum *clone HB3 as an assay performance control.

The dishes were incubated at 37°C for 1 h, with gentle mixing at 10 min intervals. Unbound cells were removed by gentle washing seven times with RPMI 1640 supplemented with D-glucose. Bound cells were fixed with 1% v/v gluteraldehyde for 1 h and stained with 10% Giemsa. For example see Figure 1c. Using an inverted light microscope at × 300 magnification images from ten consecutive non-overlapping fields for each protein and PBS treated area (six areas each per patient sample) were captured. This was equivalent to an area of 0.135 mm^2^. The number of bound infected cells/0.135 mm^2^/protein or control were counted. The results were expressed as the number of infected cells (IE) bound/mm^2 ^to each of the proteins or control [IE/mm^2 ^= (1/0.0135) × mean number of bound IE per field]. Significant binding of *P. falciparum *clone HB3 to all three test proteins compared with PBS was required in the assay performance control to validate the results for each patient isolate. Purified protein binding assays on primary field isolates are quantifiable within but not between isolates because of variability in parasitaemia. Significant binding to each protein compared with binding to PBS was determined using the Mann-Whitney *U *Test (Graphpad PRISM version 4.0a San Diego California, USA).

## Results

Five patients with PCR confirmed single species *P. knowlesi *infections were recruited into the study. Parasitaemia ranged from 0.4 to 7%. Clinical and parasitological data are summarised in Table 1. Apart from patient (P0009) with high parasitaemia and renal failure the patients had acute uncomplicated malaria. Blood samples were cultured *ex vivo *for up to 14.5 h (mean 11.3 ± 1.9 h) to allow parasite maturation and increase the relative proportion of mature trophozoite and schizont-infected red blood cells to at least 50% (mean 65.8 ± 17.51%) of all parasite stages present in preparation for binding assays (Table 1).

**Table 1:** Patient demographic, clinical and laboratory features including *ex vivo *parasite development in preparation for static binding assays

	Patient details				
**Study reference**	**P0002**	**P0006**	**P0009**	**P0010**	**P0011**
**Age**	16	47	28	27	37
**Sex**	M	F	M	M	M
**% Parasitaemia**	0.4	0.8	7	0.8	1.3
**Fever duration (days)**	7	5	6	5	4
**Axillary Temp°C**	37.5	38.5	39.2	38.7	38
**Mean Art BP mmHg**	79	96	70	86	77
**Pulse per min**	62	96	96	103	94
**Haemoglobin g/dL**	13.4	13.2	12.3	14.1	12.8
**Platelets/uL**	93,000	106,000	19,000	39,000	65,000
**White cells/uL**	6600	6700	6900	7500	4600
**Sodium mmol/L**	133	131	120	132	131
**Potassium mmol/L**	3.7	4.9	3.6	3.3	3.9
**Chloride mmol/L**	100	99	92	101	100
**Total bilirubin umol/L**	20.6	15.7	38.4	9.7	48.9
**Conjugated bilirubin umol/L**	5	12.8	23	4	32.6
**Alkaline phosphatase U/L**	100	62	87	131	233
**Alanine aminotransferase U/L**	28	21	43	35	68
**Asparagine aminotransferase UL**	17	30	118	23	22
**Blood urea mmol/L**	4.4	5.7	44.7	6	8.8
**Serum creatinine umol/L**	-	78	600	59	133
	**Static binding assay details**				
**% Mature Troph/Schizonts before *ex vivo *culture**	25	40	17	10	10
**Hours *ex-vivo *culture**	10	14.5	10	10	12
**% Mature Troph/Schizonts at start of assay**	75	50	91	63	50

### Binding to ICAM-1, VCAM and CD36

**Figure 2:**
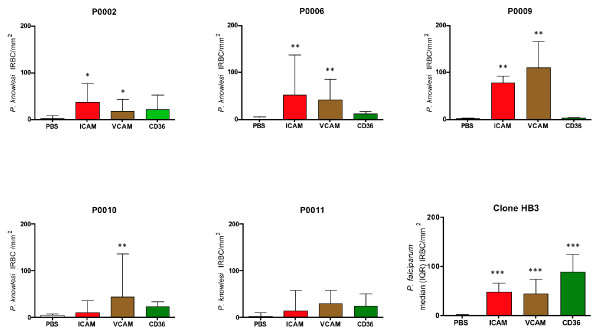
**Infected red cell binding (IRBC) to purified ICAM-1, VCAM and CD36 compared with binding to intra assay PBS controls for *P. knowlesi *infected patients P0002, P0006, P0009. P0010 and P0011**. The median and inter quartile range (IQR) of six replicates per protein or PBS control per isolated are shown. The performance character of the assay is represented by *P. falciparum *clone HB3 showing the median (IQR) binding of all control assays (15 replicates per protein and PBS). Significance of binding was analaysed using the Mann-Whitney *U *test comparing specific binding of IRBC/mm^2 ^to each purified protein with intra assay binding to PBS control areas: * *p *= < 0.05; ** *p *= < 0.01; ***p = < 0.0001.

Three (60%) isolates bound significantly to ICAM-1 and VCAM. One (20%) isolate bound significantly to VCAM and one isolate did not bind significantly to ICAM-1, VCAM or CD36 (Figure 2). None of the *P. knowlesi *isolates tested showed significant binding to CD36 (Figure 2). The performance of each binding assay was monitored using *P. falciparum *clone HB3. Clone HB3 significantly bound to ICAM-1, VCAM and CD36 in all assay plates and clone HB3 binding characteristics are summarised in Figure 2.

## Discussion

*Plasmodium knowlesi *infected erythrocytes from human infections bind in a specific but variable manner to the human endothelial cell receptors ICAM-1 and VCAM but not to CD36. Specific binding of infected erythrocytes to endothelial cell receptors is responsible for cytoadherence and, therefore, sequestration of late trophozoite and schizont infected erythrocytes from the peripheral blood circulation in *P. falciparum *malaria [6]. With few exceptions only immature trophozoite stages of *P. falciparum *infected erythrocytes are found in the circulation of patients with acute uncomplicated falciparum malaria. *Plasmodium falciparum *PfEMP1 proteins predominantly bind to CD36, a constitutively expressed scavenger pattern recognition protein, and variably to ICAM-1 and other up-regulated endothelial cell receptors [8,9,20,21]. CD36 is not expressed on areas of brain endothelium where cytoadherence occurs [22] and binding to ICAM-1 on brain endothelium is implicated in the pathophysiology of severe falciparum malaria with coma [8,9,20]. Particular expression of PfEMP1 variants, with differential ability to bind ICAM-1, has been demonstrated in malaria patients with coma [22,23]. Parasite sequestration through cytoadherence in *P. falciparum *infections can be organ specific but it is not clear yet whether this equates with virulence rather than with available binding sites and parasite binding affinity [20,21,24,25]. There is some data to suggest stratification of the *var *type early in infection and it has been suggested that cytoadherence to available receptors is responsible for this [26]. Mature stage parasites are observed in the circulation of all other types of malaria of humans, including *P. knowlesi *malaria. However, this does not exclude the possibility of a degree of parasite sequestration by specific binding to endothelial cell receptors or by other means. ICAM-1 is expressed in low copy number in resting endothelium and is up-regulated in inflammation and infection [25,27,28] including severe malaria with coma [7]. In falciparum malaria TNF has been implicated in ICAM-1 up-regulation although infected red blood cells alone are sufficient to produce this effect [25]. *Plasmodium falciparum *isolates from patients with differing degrees of disease severity show endothelial receptor binding diversity [29] with binding to ICAM-1 highest and most robust in *P. falciparum *isolates from patients with severe malaria with coma [22,29]. *Plasmodium knowlesi *proteins expressed on infected erythrocytes do not appear to bind to CD36 is this study. This lack of adhesion in a robust manner to constitutively expressed abundant endothelial receptors, such as CD36, would explain the presence of mature stage parasites in the circulation when inducible target receptors are not up-regulated. Significant but variable binding of *P. knowlesi *infected erythrocytes from human infections to the inducible endothelial receptors ICAM-1 and VCAM was demonstrated here. This result suggests that, if up-regulated on brain endothelium, *P. knowlesi *infected erythrocytes could potentially cytoadhere to ICAM-1 in that compartment. Notwithstanding this possibility, immunohistochemistry failed to detect ICAM-1 on the endothelial surfaces in parasite congested brain sections of a fatal case of severe knowlesi malaria without coma [14]. There may be technical explanations for the failure to detect ICAM-1 including limited available sections, delayed post-mortem sampling and loss of tissue integrity. It is also possible that ICAM-1 is not induced on brain endothelium in knowlesi infections and that specific binding *ex vivo *is not associated with parasite virulence in this species. The intense accumulation of infected erythrocytes observed in brain sections in fatal knowlesi malaria may have occurred through processes other than specific cytoadherence, for example infected cell agglutination [15]. This study and clinical descriptions of severe and fatal knowlesi malaria suggest that up-regulation of ICAM-1 observed in *P. falciparum *infections is less evident in *P. knowlesi *malaria and that direct or indirect induction of ICAM-1 may be a defining Plasmodium species-specific virulence factor in severe malaria with coma.

## Conclusions

In summary, *P. knowlesi *infected erythrocytes from human subjects can bind to the inducible endothelial receptors ICAM-1 and VCAM. Further work will be required to define the pathological implication of these interactions.

## Competing interests

The authors declare that they have no competing interests.

## Authors' contributions

The study was conceived by JCS and designed by JCS, AGC, SK and BS. The assays were performed by FAF with field support from AS, MAH and LCW. The manuscript was prepared by JCS, ACG, and SK. All authors had the opportunity to read and approve the manuscript.

## References

[B1] Brewster DR Kwiatkowski D White NJ Neurological sequelae of cerebral malaria in children Lancet 1990 336 1039 1043 10.1016/0140-6736(90)92498-7 1977027

[B2] Mishra SK Wiese L Advances in the management of cerebral malaria in adults Curr Opin Neurol 2009 22 302 307 10.1097/WCO.0b013e32832a323d 19434799

[B3] Aikawa M Iseki M Barnwell JW Taylor D Oo MM Howard RJ The pathology of human cerebral malaria Am J Trop Med Hyg 1990 43 30 37 2202227 10.4269/ajtmh.1990.43.30

[B4] Silamut K Phu NH Whitty C Turner GD Louwrier K Mai NT Simpson JA Hien TT White NJ A quantitative analysis of the microvascular sequestration of malaria parasites in the human brain Am J Pathol 1999 155 395 410 10.1016/S0002-9440(10)65136-X 10433933 PMC1866852

[B5] Udomsangpetch R Pipitaporn B Krishna S Angus B Pukrittayakamee S Bates I Suputtamongkol Y Kyle DE White NJ Antimalarial drugs reduce cytoadherence and rosetting *Plasmodium falciparu* J Infect Dis 1996 173 691 698 10.1093/infdis/173.3.691 8627034

[B6] Su XZ Heatwole VM Wertheimer SP Guinet F Herrfeldt JA Peterson DS Ravetch JA Wellems TE The large diverse gene family var encodes proteins involved in cytoadherence and antigenic variation of *Plasmodium falciparu*-infected erythrocytes Cell 1995 82 89 100 10.1016/0092-8674(95)90055-1 7606788

[B7] Turner GD Morrison H Jones M Davis TME Looareesuwan S Buley ID Gatter KC Newbold CI Pukritayakamee S Nagachinta B White NJ Berendt AR An immunohistochemical study of the pathology of fatal malaria. Evidence for widespread endothelial activation and a potential role for intercellular adhesion molecule-1 in cerebral sequestration Am J Pathol 1994 145 1057 1069 7526692 PMC1887431

[B8] Udomsangpetch R Taylor BJ Looareesuwan S White NJ Elliott JF Ho M Receptor specificity of clinical *Plasmodium falciparu *isolates: nonadherence to cell-bound E-selectin and vascular cell adhesion molecule-1 Blood 1996 88 2754 2760 8839872

[B9] McCormick CJ Craig A Roberts D Newbold CI Berendt AR Intercellular adhesion molecule-1 and CD36 synergize to mediate adherence of *Plasmodium falciparu*-infected erythrocytes to cultured human microvascular endothelial cells J Clin Invest 1997 100 2521 2529 10.1172/JCI119794 9366566 PMC508452

[B10] Berendt AR Simmons DL Tansey J Newbold CI Marsh K Intercellular adhesion molecule-1 is an endothelial cell adhesion receptor for *Plasmodium falciparu* Nature 1989 341 57 59 10.1038/341057a0 2475784

[B11] Cox-Singh J Davis TM Lee KS Shamsul SS Matusop A Ratnam S Rahman HA Conway DJ Singh B *Plasmodium knowles *malaria in humans is widely distributed and potentially life threatening Clin Infect Dis 2008 46 165 171 10.1086/524888 18171245 PMC2533694

[B12] Daneshvar C Davis TM Cox-Singh J Rafa'ee MZ Zakaria SK Divis PC Singh B Clinical and laboratory features of human *Plasmodium knowles *infection Clin Infect Dis 2009 49 852 860 10.1086/605439 19635025 PMC2843824

[B13] William T Menon J Rajahram G Chan L Ma G Donaldson S Khoo S Frederick C Jelip J Anstey NM Yeo TW Severe *Plasmodium knowles *Malaria in a Tertiary Care Hospital, Sabah, Malaysia Emerg Infect Dis 2011 17 1248 1255 10.3201/eid1707.101017 21762579 PMC3381373

[B14] Cox-Singh J Hiu J Lucas SB Divis PC Zulkarnaen M Chandran P Wong KT Adem P Zaki SR Singh B Krishna S Severe malaria - a case of fatal *Plasmodium knowles *infection with post-mortem findings: a case report Malar J 2010 9 10 10.1186/1475-2875-9-10 20064229 PMC2818646

[B15] Howard RJ Barnwell JW Kao V Antigenic variation of *Plasmodium knowles *malaria: identification of the variant antigen on infected erythrocytes Proc Natl Acad Sci USA 1983 80 4129 4133 10.1073/pnas.80.13.4129 6191331 PMC394214

[B16] al-Khedery B Barnwell JW Galinski MR Antigenic variation in malaria: a 3' genomic alteration associated with the expression of a *P. knowles *variant antigen Mol Cell 1999 3 131 141 10.1016/S1097-2765(00)80304-4 10078196

[B17] Korir CC Galinski MR Proteomic studies of *Plasmodium knowles *SICA variant antigens demonstrate their relationship with *P. falciparu *EMP1 Infect Genet Evol 2006 6 75 79 10.1016/j.meegid.2005.01.003 16376842

[B18] Milner DA Jr Rethinking cerebral malaria pathology Curr Opin Infect Dis 2010 23 456 463 10.1097/QCO.0b013e32833c3dbe 20613511

[B19] Singh B Kim Sung L Matusop A Radhakrishnan A Shamsul SS Cox-Singh J Thomas A Conway DJ A large focus of naturally acquired *Plasmodium knowles *infections in human beings Lancet 2004 363 1017 1024 10.1016/S0140-6736(04)15836-4 15051281

[B20] Udomsangpetch R Reinhardt PH Schollaardt T Elliott JF Kubes P Ho M Promiscuity of clinical *Plasmodium falciparu *isolates for multiple adhesion molecules under flow conditions J Immunol 1997 158 4358 4364 9126999

[B21] Tse MT Chakrabarti K Gray C Chitnis CE Craig A Divergent binding sites on intercellular adhesion molecule-1 (ICAM-1) for variant *Plasmodium falciparu *isolates Mol Microbiol 2004 51 1039 1049 10.1046/j.1365-2958.2003.03895.x 14763979

[B22] Ochola LB Siddondo BR Ocholla H Nkya S Kimani EN Williams TN Makale JO Liljander A Urban BC Bull PC Szestak T Marsh K Craig AG Specific receptor usage in *Plasmodium falciparu *cytoadherence is associated with disease outcome PLoS One 2011 6 e14741 10.1371/journal.pone.0014741 21390226 PMC3048392

[B23] Janes JH Wang CP Levin-Edens E Vigan-Womas I Guillotte M Melcher M Mercereau-Puijalon O Smith JD Investigating the host binding signature on the *Plasmodium falciparu *PfEMP1 protein family PLoS Pathog 2011 7 e1002032 10.1371/journal.ppat.1002032 21573138 PMC3088720

[B24] Montgomery J Mphande FA Berriman M Pain A Rogerson SJ Taylor TE Molyneux ME Craig A Differential var gene expression in the organs of patients dying of falciparum malaria Mol Microbiol 2007 65 959 967 10.1111/j.1365-2958.2007.05837.x 17617167 PMC2170262

[B25] Wu Y Szestak T Stins M Craig AG Amplification of *P. falciparu *cytoadherence through induction of a pro-adhesive state in host endothelium PLoS One 2011 6 e24784 10.1371/journal.pone.0024784 22043276 PMC3197193

[B26] Lavstsen T Magistrado P Hermsen CC Salanti A Jensen AT Sauerwein R Hviid L Theander TG Staalsoe T Expression of *Plasmodium falciparu *erythrocyte membrane protein 1 in experimentally infected humans Malar J 2005 4 21 10.1186/1475-2875-4-21 15857512 PMC1112614

[B27] McGuire W Hill AV Greenwood BM Kwiatkowski D Circulating ICAM-1 levels in falciparum malaria are high but unrelated to disease severity Trans R Soc Trop Med Hyg 1996 90 274 276 10.1016/S0035-9203(96)90244-8 8758074

[B28] Turner GD Ly VC Nguyen TH Tran TH Nguyen HP Bethell D Wyllie S Louwrier K Fox SB Gatter KC Day NP Tran TH White NJ Berendt AR Systemic endothelial activation occurs in both mild and severe malaria. Correlating dermal microvascular endothelial cell phenotype and soluble cell adhesion molecules with disease severity Am J Pathol 1998 152 1477 1487 9626052 PMC1858439

[B29] Newbold C Warn P Black G Berendt A Craig A Snow B Msobo M Peshu N Marsh K Receptor-specific adhesion and clinical disease in *Plasmodium falciparu* Am J Trop Med Hyg 1997 57 389 398 9347951 10.4269/ajtmh.1997.57.389

